# Deep-sea anthropogenic macrodebris harbours rich and diverse communities of bacteria and archaea

**DOI:** 10.1371/journal.pone.0206220

**Published:** 2018-11-28

**Authors:** Lucy C. Woodall, Anna D. Jungblut, Kevin Hopkins, Andie Hall, Laura F. Robinson, Claire Gwinnett, Gordon L. J. Paterson

**Affiliations:** 1 Department of Life Sciences, The Natural History Museum, London, United Kingdom; 2 Nekton Foundation, Begbroke Science Park, Oxford, United Kingdom; 3 Department of Zoology, University of Oxford, South Parks Road, Oxford, UK, United Kingdom; 4 School of Earth Sciences, University of Bristol, Bristol, United Kingdom; 5 Department of Criminal Justice and Forensic Science, Staffordshire University, Stoke-On-Trent, United Kingdom; University of Milan, ITALY

## Abstract

The deep sea is the largest biome on earth, and microbes dominate in biomass and abundance. Anthropogenic litter is now almost ubiquitous in this biome, and its deposition creates new habitats and environments, including for microbial assemblages. With the ever increasing accumulation of this debris, it is timely to identify and describe the bacterial and archaeal communities that are able to form biofilms on macrodebris in the deep sea. Using 16S rRNA gene high throughput sequencing, we show for the first time the composition of bacteria and archaea on macrodebris collected from the deep sea. Our data suggest differences in the microbial assemblage composition across litter of different materials including metal, rubber, glass, fabric and plastic. These results imply that anthropogenic macrodebris provide diverse habitats for bacterial and archaeal biofilms and each may harbour distinct microbial communities.

## Introduction

The accumulation of marine debris is a growing challenge that is seen across the globe including in some of the most remote locations (e.g. polar regions and the deep sea) [1]. Researchers are still working to establish the risk to the environment, particularly from the plastic component. However some of the realised and hypothesised impacts are those that cause physical (e.g. entanglement), chemical (e.g. bioaccumulation) or biological (e.g. invasive species) harm [2]. Until recently the colonisation of debris by microbes had not been considered as a risk [3], and although data are still not extensive there is evidence for some specific colonisation patterns on plastics that have leaked into the environment [4].

Laboratory incubation studies, designed to emulate marine conditions, showed that litter including plastic, metal and glass, can be colonised within days [5, 6] with the physical composition, surface texture and history of the litter affecting the formation and subsequent succession of biofilm communities [7, 8]. Diverse microbial assemblages have also been found in biofilms from plastic debris collected from ocean surface water [3, 4, 9–11], the shallow coastal seabed [12] and intertidal zones [13]. Most of these studies have investigated plastic suspended in the water column, with few exploring the litter fallen to the sea floor [12], and all have limited their research to items made from plastic. Some studies have indicated that Alpha- and Gammaproteobacteria and Bacteroidetes are common on plastic debris [3, 4] and that polymer type can affect the taxa colonizing the debris, but this has not been seen in all studies [6]. Furthermore, extensive sampling of Australian waters, and the Atlantic and Pacific Oceans showed a consistently high diversity of microbial colonizers on floating plastic litter, which varied depending on geographic location [9, 14]. All studies reported patchiness and distinctiveness of the assemblage composition associated with plastic items/fragments.

In this study we document and compare the bacterial and archaeal communities on deep-sea macrodebris comprised of a wide range of materials including metal, rubber, glass, fabric and plastic.

## Methods

### Sample collection and preservation

No permits were required as all locations are in the high seas, outside any jurisdiction. No endangered or protected species were involved in this study. A total of 12 litter items and two sediment cores were collected during research cruise JC94 (October-December 2013), aboard R.R.S. *James Cook* (Table 1). All sampling was conducted using remotely operated vehicle (ROV) *Isis* [15]. ROV dives were made on submarine geomorphological features in the equatorial Atlantic Ocean (seamounts and ridges), encompassing both eastern and western basins. Litter items were stored in bioboxes on the ROV, until vehicle recovery back to the ship. Once on deck, litter samples were moved to a ship laboratory using sterile metal forceps and a sterile metal container, and sediment cores were transported in enclosed core tubes. Sediment cores were collected using anti-contamination procedures [16] and were sliced, using a sterile metal plate, to 2 cm depth. Litter items were wrapped in three layers of sterile metal foil, placed in individual cardboard boxes and stored immediately at -80°C.

**Table 1:** Summary of sample details, including location, water depth, site, ocean basin, material, morphology and type.

Sample	Latitude	Longitude	Water Depth (m)	Site	Basin	Material	Morphology	Type
Deep-East	5° 37.51’ N	26° 57.70’ W	1000	Knipovich	East	Sediment	n/a	Sediment
Deep-West	14° 53.63’ N	48° 7.39’ W	1050	Vayda	West	Sediment	n/a	Sediment
Plastic 1	14° 52.81’ N	48° 8.39’ W	410	Vayda	West	Polyvinylchloride	Fragment irregular	Litter
Plastic 2	14° 52.77’ N	48° 8.39’ W	410	Vayda	West	Polyethylene	Flatten braided twine	Litter
Plastic 3	14° 52.70’ N	48° 8.35’ W	400	Vayda	West	Polyethylene	Twisted twine	Litter
Plastic 4	14° 52.70’ N	48° 8.35’ W	390	Vayda	West	Polyethylene	Flatten braided twine	Litter
Plastic 5	14° 52.70’ N	48° 8.35’ W	390	Vayda	West	Polyamide	Cylindrical line	Litter
Metal 1	5° 37.61’ N	26° 56.67’ W	640	Knipovich	East	Steel	Cylinder	Litter
Fabric 1	9° 14.80’ N	21° 19.68’ W	200	Carter	East	Polyester	Flat irregular fragment	Litter
Rubber 1	9° 14.69’ N	21° 19.56’ W	270	Carter	East	Unknown	Cylindrical ring	Litter
Rubber 2	9° 12.95’ N	21° 18.36’ W	1180	Carter	East	Unknown*	Flat square with cut sections	Litter
Rubber 3	10° 44.39’ N	44° 33.67’ W	1120	Vema	West	Unknown*	Irregular cylindrical length	Litter
Glass 1	5° 35.65’ N	26° 58.60’ W	2320	Knipovich	East	Glass	Bottle shaped	Litter
Glass 2	10° 46.85’ N	44° 35.93’ W	2950	Vema	West	Glass	Bottle shaped	Litter

* denotes samples that were different from each other, but their actual material could not be determined.

### Litter characteristics

Plastic, rubber and fabric samples were subjected to Fourier Transform Infrared Spectroscopy–Attenuated Total Reflectance (FTIR–ATR) [17] after the genetic sampling was complete. The FTIR utilised was a Thermo Nicolet, Avatar 370 spectrometer with a Specac Golden Gate, single reflection diamond ATR with a ZnSe focussing element, run with OMNIC software. Before each sample was analysed a background reading was made. The test was set up for 32 scans with a resolution of four. For each sample, the major peaks between 500 cm^-1^ and 4000 cm^-1^ were analysed in order to identify polymer type. Plastic 1–4 and Fabric 1 were placed directly upon the diamond cell and the golden gate apparatus used to flatten the sample against the sampling window. Due to the shape and debris present upon the outer layer of Plastic 5 and Rubbers 1–3, the sample had to be subsampled and the outer debris removed with a scalpel. These smaller pieces were then handled in the same manner as Plastic 1–4 and Fabric 1.

### Genetic analysis

On shore at the Natural History Museum laboratories, litter items were removed from storage, allowed to defrost in a sterile environment and then surfaces were sampled by wiping sterile swabs directly on the items five times with a stroke of approx. 5 cm. A tube with sterile water was left open to the air and next to the sampling station to act as an environmental control while litter items were exposed to the air. From each sediment core, four 4 g subsamples were taken from the 0–2 cm horizon, environmental controls were deployed next to the sampling station (tube with sterile water), and all work was conducted using sterile equipment. Swab tips were cut off into sterile tubes, and DNA extracted from the swab tips, deep-sea sediment, and environmental controls using PowerBiofilm^TM^ and PowerSoil^TM^ DNA isolation extraction kits (MoBio Laboratories, Carlsbad, CA, USA), following the methods suggested by the manufacturer. To check for bacterial and archaeal DNA, all samples were amplified for the V4 region of 16S rRNA using 515F/806R primers [18]. The 25 μl PCR consisted of 12.7 μl PCR Water, 5 μl 5X Buffer, 1 μl BSA 10 mg/ml (Promega, Maidson WI, USA), 2.5 μl MgCl_2_ (25 mM), 1.25 μl of each (10 μM) forward and reverse primer, 0.2 μl dNTPs (100 m, Bioline, London, UK), 0.1 μl GoTaq (Promega) and 1 μl DNA, and was subjected to the following thermocycle: initial 3 mins at 94°C, then 45 s at 94°C, 60 s at 50°C and 90 s at 72°C for 30 cycles with a final extension of 10 mins at 72°C. An electrophoresis gel (1%) showed all environmental controls did not contain amplifiable DNA and were excluded from further processing. Sample DNA was amplified in triplicate 25 μl reactions, varying the amount of DNA added to each replicate (0.5 μl, 1.0 μl and 1.5 μl), using tagged V4 region primers including sample specific tags. Amplicons were cleaned using AxyPrep PCR cleanup beads (Axygen, Corning, NY, USA) following the manufacturer’s instructions. The cleaned amplicon libraries were quantified using a Qubit dsDNAHS Assay Kit (Invitrogen, Carlsbad, CA, USA) and were pooled at equal concentrations. Paired-end sequencing (2 x 300 bp) was performed on an Illumina Miseq platform at Natural History Museum Sequencing facility following the protocol as outlined by the Earth Microbiome Project [18].

### High throughput sequence processing and statistical analyses

Using the QIIME toolkit v1.9.1 [19] sequences were quality filtered using recommended default parameters for QIIME [20] and assigned to Operational Taxonomic Units (OTUs) using open reference picking and the UCLUST algorithm [21] at 97% similarity. A representative sequence list was created and taxa assigned using GreenGenes v13.8 [22] OTU database. Chimeras were identified with USEARCH 6.1 algorithm and removed. Singleton OTUs, mitochondrial and chloroplast sequences were also filtered out using the script ‘filter_taxa_from_otu_table.py’ and data summarized for all taxonomic levels including for OTUs. Finalized OTUs are deposited as PRJNA496502 SRA NCBI raw reads accession number, all subsequent analyses were performed in PRIMER v7 [23]. OTUs were rarefied to 26,000 in QIIME, to standardize data. Rarifying was the simplest approach considering the characteristics of our libraries [24]. For beta diversity, Bray-Curtis was used to estimate dissimilarity. No transformations were conducted as assemblages comprised a large number of rare taxa and these were considered important for appropriate ecological interpretation of results. The Venn diagram to visualise similarity across litter biofilm OTUs was drawn using http://bioinformatics.psb.ugent.be/webtools/Venn/. Using PRIMER, similarities were visualised with CLUSTER analysis and statistical support was shown using the Similarity Profile Routine (SIMPROF) test. Multivariate Analysis of Similarity (ANOSIM) was conducted to compare assemblages between basins (Table 1) using 999 random permutations. Due to the low number of OTUs assigned to a reference lower than class, even though singleton OTUs were removed, comparisons were limited to OTU, class and phylum levels.

## Results

### Sample characteristics

**Fig 1:**
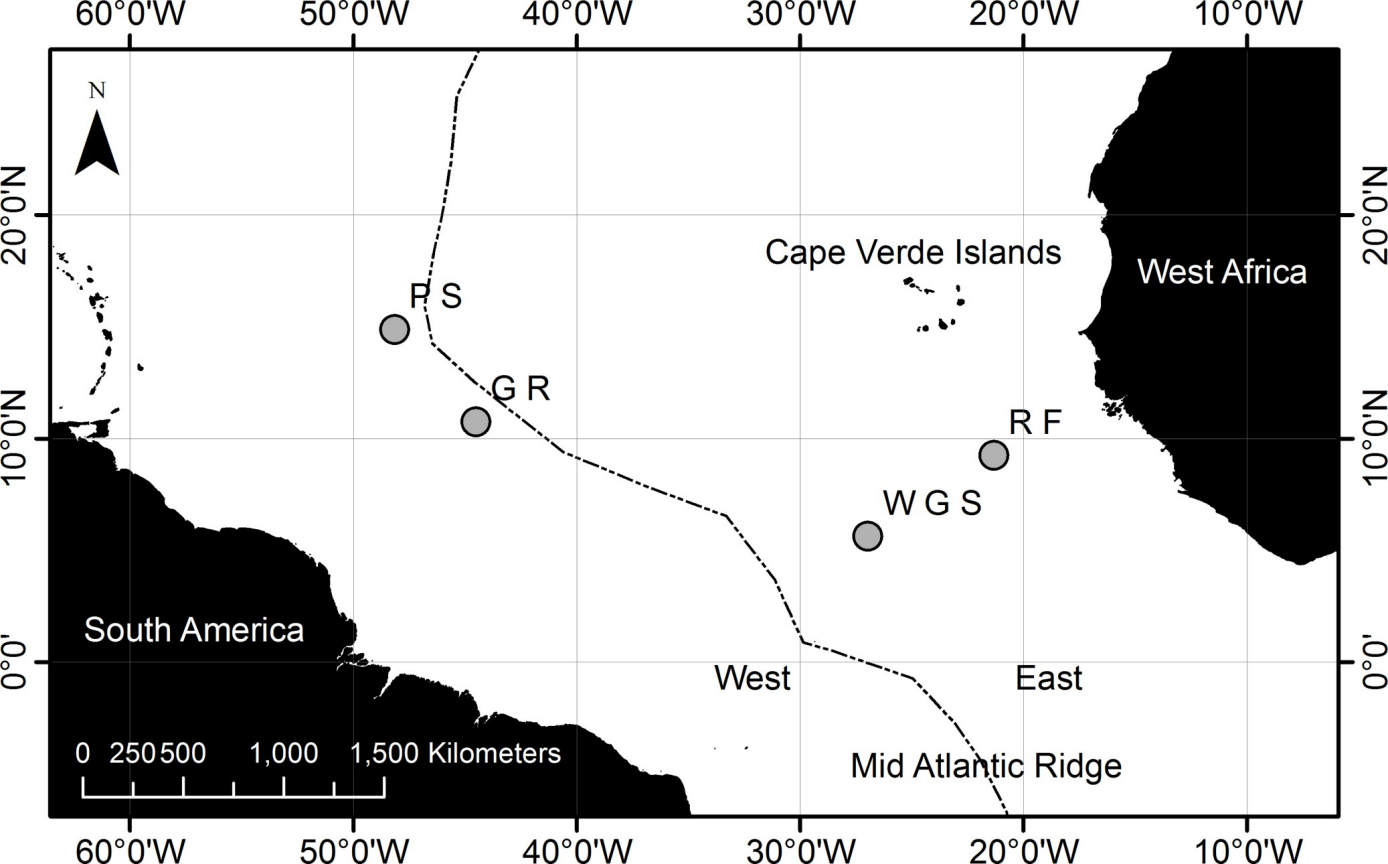
Map to show sampling locations in the equatorial Atlantic. Samples types are represented as S = Sediment, P = Plastic, M = Metal, F = Fabric, R = Rubber and G = Glass.

Twelve anthropogenic litter items comprising metal, rubber, glass, fabric and plastic, were collected from four Atlantic Ocean sites (Fig 1). The litter items showed some differences in morphology ranging from cylindrical line to braided twine, and fully intact items (bottles) to small fragments (Table 1). They were made from nine different materials/polymers. Five of the plastics were identified as polyethylene, however each produced different overall spectra, and therefore can be differentiated from each other. It was not possible to identify specific rubber type due to poor quality spectra as a result of uneven surface morphology and due to the presence of surface debris that could not be completely removed. However, there were sufficient peaks in the spectra of Rubber 2 and 3 (Table 1) to differentiate these from one another in terms of their composition, indicating that Rubber 2 and 3 were made of different chemicals. There was insufficient information for Rubber 1 to allow comparison with Rubber 2 and 3. As there were only total of twelve samples, the sample number was too small to permit comparison between and among samples of the same polymer or items with similar morphologies. Both sediment samples contained microplastic fibres, but no large fragments of plastic. Microbial analysis of the microplastics removed from the sediment samples was not successful, as insufficient DNA was extracted.

### Bacterial and archaeal composition of litter biofilms and sediment

A total of 3,993,833 16S rRNA sequences, were obtained from the 12 biofilm and two sediment samples (Fig 1, Table 1), which were clustered into a total of 29,856 Operational Taxonomic Units (OTUs) at 97% similarity level, after quality control and removal of mitochondrial and chloroplast sequences and singletons.

**Fig 2:**
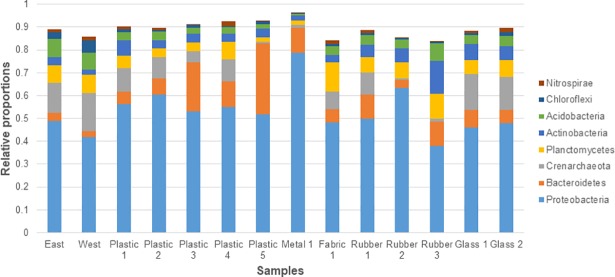
Relative proportions (%) of bacterial and archaeal phyla. Bars show a comparison of the relative proportions of the eight most abundant phyla across all samples.

The microbial OTUs grouped into 54 different phyla, 277 known families and 344 known genera. The eight phyla of greatest relative abundance, each were >1% of the total OTUs (Fig 2), with the other 46 together making up 7% of the OTUs. The most abundant bacterial phylum was Proteobacteria which accounted for >52% of total OTUs, followed by Bacteroidetes (10%), and Crenarchaeota, the only archaea phylum present in all samples, had a relative abundance of 9%.

**Fig 3:**
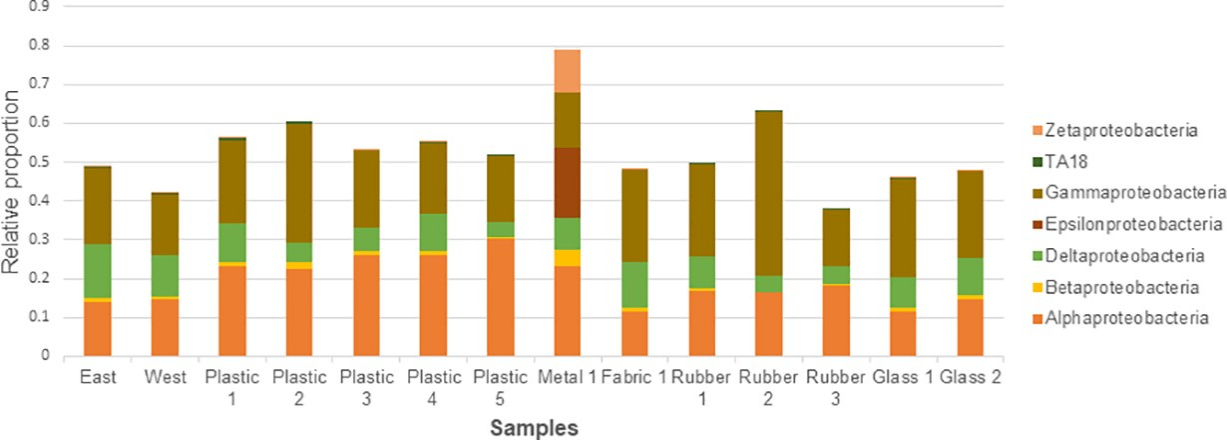
Relative proportions (%) of proteobacteria across all samples.

Of the eight most common phyla found in all samples, only 20% (10.5–36.9) of OTUs were assigned to recognised genera, thus no further analysis of the level was conducted. At phylum level, variation in assemblages between all samples was seen, but sediment and glass were most similar (Fig 2). Metal had highest relative abundance of Proteobacteria. The biofilm composition data revealed that Bacteroidetes was more abundant on litter than in the sediment (Fig 2). Relative abundance of Actinobacteria was an order of magnitude greater (14.6%) on Rubber 3 than the other samples. Glass and sediment biofilms had the largest proportion of Crenarchaeota. Within Proteobacteria, the composition at class level was similar across most samples (Fig 3), however the metal sample had a much higher proportion of Epsilonproteobacteria and was the only sample where Zetaproteobacteria was seen.

**Fig 4:**
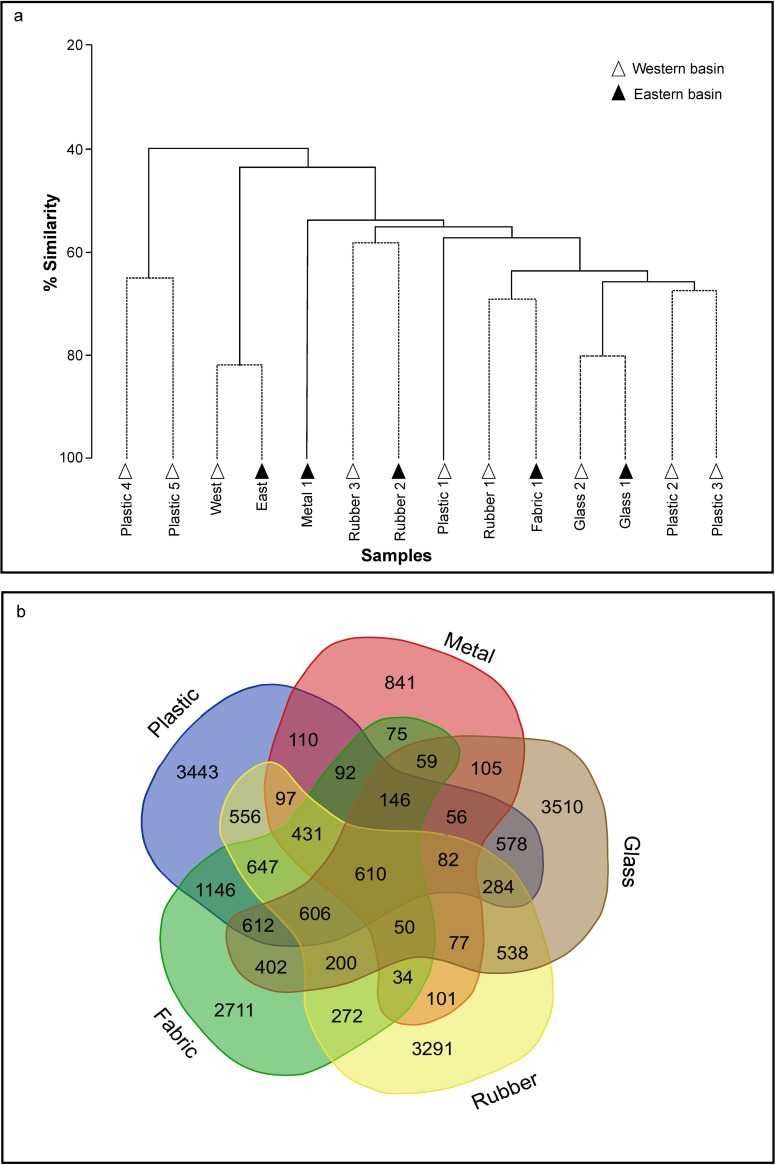
Comparisons of litter and sediment microbial assemblages. (a) Bray-Curtis similarity based on CLUSTER analysis and SIMPROF test identifies eight coherent groups which are statistically distinct at the 5% level (solid lines). The dash line shows clusters that have no statistical support. (b) Venn diagram showing the number of shared OTUs between litter biofilms.

The cluster analysis shows eight groups that are distinct from each other (Fig 4A), these groups do not always originate from the same material or basin. Most OTUs were only present on a single material type (Fig 4B), and just 0.3% of OTUs were recorded from all samples, suggesting high beta diversity. Comparison of the litter and sediment samples identified that litter samples differed from the sediment samples and by type of macrodebris [1-way ANOSIM R = 0.584, p < 0.01]. These differences derive from both the relative abundance and composition, but no obvious pattern in assemblage composition or key set of OTU was observed across litter types (S1 Table). Using a significance level of 0.01, no significant difference was determined between basins (east and west) [1-way ANOSIM R = 0.117, p = 0.12].

H’ (Shannon-Wiener Index) and OTU richness (Margalef) were consistent across most sample types (Table 2). Evenness (Pielou) was similarly low across most assemblages, however, the biofilm from the single metal item was lower for all parameters of diversity, richness and evenness.

**Table 2:** Summary of data (Sample number (n), Number of raw sequences reads (R), Number of OTUs (O)) and diversity indices (Shannon’s diversity (H’), Margalef’s richness (d), Pielou’s evenness (J’)).

Sample	n	R	O	H’ (log e)	d	J’
Sediment	2	775699	15700	7.27	1282	0.752
East	1	173630	10482	7.24	907	0.782
West	1	602069	10636	7.04	920	0.759
Litter	12	3218134	21656	7.22	1628	0.723
Plastic	5	753628	9494	6.67	768	0.728
Metal	1	569070	2966	4.39	273	0.549
Fabric	1	438431	7912	7.33	728	0.817
Rubber	3	1098692	7876	6.27	659	0.699
Glass	2	358313	8093	6.83	701	0.759

## Discussion

Our data indicate that all anthropogenic macrodebris, not just plastic items, are potentially important microbial habitats in the deep sea. We have demonstrated that biofilm assemblages on deep-sea litter appear to vary across different litter material (metal, rubber, glass, fabric and plastic). The ‘Plastisphere’ has already been coined as a descriptive word for the habitat provided by plastics in the marine environment [3, 9, 12]. Our data indicate that all anthropogenic debris harbours microbial assemblages and therefore propose an extension of the ‘Plastisphere’ to the ‘Litterosphere’.

The results suggest a similar microbial richness across all samples (sediment and litter), which is contrary to results from floating microplastic collected in surface water [9], and from shallow benthic microplastic from coastal environments [12]. In these previous studies, plastic microbial richness was less than the surrounding environment. Our litter microbial assemblages were dominated by a few abundant taxa, and a large number of OTUs recorded by only a few sequences. This pattern is common to many other taxa in the deep sea [24].

The litter bacterial and archaeal communities investigated here share some OTUs with local deep-sea sediment, but could have also drawn taxa from surrounding water, or other natural substrates such as rock, fauna and dead skeletons of benthic calcifiers such as corals, and even could have been colonised at the surface or in the water column. Further work is required on co-located litter water and substrata from the deep sea to fully determine the origin of taxa colonising litter, and understand assemblages changes during succession. There is a recognised microbial succession shown on marine plastic litter in shallow water [25]. As it is not possible to determine how long our litter items were on the seabed, or more broadly, their history since release into the environment, the community differences seen could be as a result of different stages of succession. Although possible, due the remote nature of the sampling locations and observed signs of weathering it is unlikely that the litter items sampling here were recently leaked into the marine environment.

Our study suggests that there are differences between the bacteria and archaea of litter items of different materials. Given the challenges of collecting experiments in the deep-sea, it is not yet possible to provide replicate analyses for each substrate type. Our sample set was limited by logistical challenges the remoteness of sampling locations. It included three different polymers, with only polyethylene represented by more than one sample, and every sample had a different shape, texture and surface morphology. Therefore with the data available we cannot determine systematically if the microbial communities differ by polymer type or morphology. However, a previous study [9] identified significant differences in microbial assemblages between polymer types suggesting that polymer type may be an important factor in determining assemblage composition and diversity. We found that bacterial and archaeal communities on metal had the lowest diversity, richness and evenness of all substrates, and the metal sample was the only one where Zetaproteobacteria were present. Zetaproteobacteria are iron-oxidizing bacteria, and are regularly found associated with metalliferous deposits [26] and corroding steel [27]. The concept of microbiologically influenced corrosion of mild steel is a well-known phenomenon [28] which are dominated by Proteobacteria, however there have been no previous specific studies on biofilm communities on metal litter. In addition natural formations rich in metal, such as ferromanganese nodules have been found to have less diverse microbial communities than surrounding sediment [29] as do sediments contaminated with metals [30]. Our observation is consistent with that finding.

The bacteria and archaea in the biofilms could fulfil a range of ecological functions. For example, almost all of the archaea are Thaumararchaeota, which act within the nitrogen cycle, to oxidize ammonia [31]. Furthermore, Proteobacteria, specifically Alpha- and Gammaproteobacteria dominate many marine ecosystems [32] and are also highly abundant in the litter biofilms. These classes of bacteria are important for carbon cycling in marine ecosystems [5, 33, 34]. In future it will be important to include microbial eukaryotes in analyses as these can have high taxa richness in marine water and biofilm habitats as well as play an important role in carbon cycling and other microbial interactions such as parasites and predators in microbial food webs [35]. Metagenomic investigations are therefore required to understand bacterial, archaeal and eukaryotic taxonomic and functional diversity of the litter biota, and differences between communities across the various types of litter and natural materials.

In summary, our new data suggest that anthropogenic litter supports biofilms with rich bacterial and archaeal assemblages in the deep sea. Microbial colonisation also appears to be linked to substrate type, and indicates that litter characteristics may play an important role in structuring of deep-sea litter biofilms. With this study we suggest that deep-sea anthropogenic debris harbours microbial assemblages and comparisons between litter biofilms and sediments (albeit based on limited sample numbers and slightly different methodology) indicate difference in the microbial flora. These results reveal that litter is providing a substratum and perhaps a substrate which is selecting for different assemblages of microbes that are present in the sedimentary environment. However, how this might affect ecosystem processes is as yet unclear but as the amount of debris increases, it will likely play an increasing role in the biosphere.

## Supporting information

S1 Table SIMPER analysis. (CSV)
